# DNA protein crosslink proteolysis repair: From yeast to premature ageing and cancer in humans

**DOI:** 10.1016/j.dnarep.2018.08.025

**Published:** 2018-11

**Authors:** John Fielden, Annamaria Ruggiano, Marta Popović, Kristijan Ramadan

**Affiliations:** a Cancer Research UK and Medical Research Council Oxford Institute for Radiation Oncology, Department of Oncology, University of Oxford, Roosevelt Drive, Oxford, OX3 7DQ, UK; b Ruđer Bošković Institute, Bijenička cesta 54, 10000 Zagreb, Croatia

**Keywords:** 5-aza-dC, 5-aza-2′-deoxycytidine, ACRC, acidic repeat containing, APC, anaphase promoting complex, ATM, ataxia-telangiectasia mutated, *C. elegans*, *Caenorhabditis elegans*, CPT, camptothecin, CtIP, C-terminal-binding protein interacting protein, ETO, etoposide, DNMT1, DNA methyltransferase 1, DPC, DNA-protein crosslink, DPCP, DNA-protein crosslink proteolysis, DSB, double-strand break, ERCC1, excision repair cross-complementing gene 1, FA, formaldehyde, GCNA, germ cell nuclear antigen, HER2, human epidermal growth factor receptor 2, HR, homologous recombination, IR, ionising radiation, MAFFT, multiple alignment using fast fourier transform, MEFs, mouse embryonic fibroblasts, MRN complex, Mre11-Rad50-Nbs1 complex, NER, nucleotide excision repair, NSCLC, non-small cell lung cancer, PARP, poly (ADP-ribose) Polymerase, PCNA, proliferating cell nuclear antigen, PIAS, protein inhibitor of activated STAT protein, PTM, post-translational modification, RJALS, Ruijs–Aalfs syndrome, *S. cerevisiae*, *Saccharomyces cerevisiae*, SHP, Src homology 2-containing Phosphatase, SIM, SUMO-interacting motif, SPRTN, SprT-like N-terminal domain, ss/ds DNA, single-stranded/double-stranded DNA, STUbL, SUMO-targeted ubiquitin ligases, SUMO, small ubiquitin-like modifier, TDP1/2, tyrosyl-DNA phosphodiesterase 1/2, TLS, translesion synthesis, Top1/2-cc, topoisomerase 1/2 cleavage complex, UBC9, ubiquitin carrier protein 9, UBZ, ubiquitin-binding zinc finger, UV, ultraviolet, WLM, Wss1p-like metalloproteases, Wss1, weak suppressor of Smt3-1, XPF, *Xeroderma Pigmentosum* group F-complementing protein, DNA-protein crosslinks, SPRTN protease, Post-translational modification, Genome stability, Ageing, Cancer

## Abstract

DNA-protein crosslinks (DPCs) are a specific type of DNA lesion consisting of a protein covalently and irreversibly bound to DNA, which arise after exposure to physical and chemical crosslinking agents. DPCs can be bulky and thereby pose a barrier to DNA replication and transcription. The persistence of DPCs during S phase causes DNA replication stress and genome instability. The toxicity of DPCs is exploited in cancer therapy: many common chemotherapeutics kill cancer cells by inducing DPC formation.

Recent work from several laboratories discovered a specialized repair pathway for DPCs, namely DPC proteolysis (DPCP) repair. DPCP repair is carried out by replication-coupled DNA-dependent metalloproteases: Wss1 in yeast and SPRTN in metazoans. Mutations in *SPRTN* cause premature ageing and liver cancer in humans and mice; thus, defective DPC repair has great clinical ramifications. In the present review, we will revise the current knowledge on the mechanisms of DPCP repair and on the regulation of DPC protease activity, while highlighting the most significant unresolved questions in the field. Finally, we will discuss the impact of faulty DPC repair on disease and cancer therapy.

## 1 Introduction

**Fig. 1:**
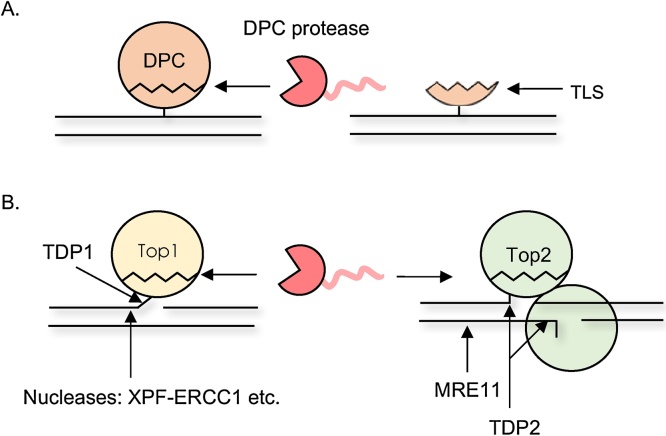
Schematic of DPC repair pathways. A. Non-enzymatic DPCs are cleaved by proteases and the DNA-bound peptide remnant is bypassed by translesion synthesis (TLS) polymerases. B. Following cleavage of the bulk of the protein component of the enzymatic DPCs, Top1- and Top2-ccs, peptide remnants can be excised by phosophodiesterases. Alternatively, nucleases can also remove DPCs by cleaving the DNA to which DPCs are attached.

DNA-protein crosslinks (DPCs) are frequent and particularly toxic DNA lesions, as they impede essential DNA transactions. They consist of a protein covalently and irreversibly bound to DNA. DPCs can arise after exposure to physical, chemical or chemotherapeutic agents and through the faulty action of certain DNA metabolizing enzymes [1]. Physical agents include ionizing radiation (IR), which target DNA and/or proteins generating reactive radicals, and UV light, which excites DNA bases that react with amino acids. Chemical crosslinking is caused by metals and aldehydes; aldehydes act by crosslinking the amino and imino groups from amino acid side chains and DNA bases to one another. Aldehydes are released during lipid peroxidation (*e.g*. malondialdehyde), histone demethylation (formaldehyde) and alcohol breakdown, implying that threats posed by DPCs are ubiquitous [2,3]; indeed, aldehydes are also present in the environment (*e.g.* cosmetics). Additional sources of DPCs are abasic sites within nucleosomes [4]. Some enzymes form a transient covalent intermediate with DNA as part of their catalytic cycle; this covalent intermediate can be stabilized in the presence of, *e.g.*, a distortion on DNA or specific poisons, resulting in an abortive reaction and trapping of the protein in a DPC. These DPCs are classified as enzymatic DPCs, as opposed to the formerly described and more general non-enzymatic DPCs [5] (Fig. 1). A renowned case of enzymatic DPC forms following exposure of Topoisomerase 1 to Topoisomerase 1-specific poisons called camptothecins (namely Top1 cleavage complex, or Top1-cc), which are widely used in cancer therapy for their cytotoxicity [6].

Mammalian cells are challenged with approximately 6000 DPCs during exponential growth [7]. The chromatin environment is generally crowded and exposed to a variety of crosslinking agents, meaning any protein in the vicinity of DNA can potentially be crosslinked to DNA. Considering their bulky nature, DPCs present major barriers, especially to DNA replication and transcription [1], ultimately causing DNA replication stress and genomic instability; therefore, DPC removal is essential for cell survival [8].

Recently, the effort of several laboratories has brought a new pathway to the attention of the DNA repair field. This repair mechanism is specific for DPCs and is carried out by replication-coupled DNA-dependent proteases in eukaryotes. DPC proteolysis repair has great medical significance since defective DPC protease activity is associated with progeria and cancer predisposition in humans and mice. We will review the current knowledge on DPC repair and discuss the regulatory principles of DPC proteases. We will present Top-cc as a prototypical DPC with significant medical implications and discuss how DPC formation is exploited in cancer therapy.

## 2 DPC proteolysis repair

Genetic and biochemical data from bacteria and yeast led to the long-standing assumption that DPC repair relies on canonical repair pathways: homologous recombination (HR) and nucleotide excision repair (NER) [9]. However, this view was challenged by the recent discovery of DPC proteolysis (DPCP) repair [8]. The existence of DPC-specific proteases was first reported by the Jentsch laboratory with the discovery of the DNA-dependent metalloprotease Wss1 (Weak Suppressor of Smt3) in *S. cerevisiae* [10]. Wss1 cleaves DNA binding proteins *in vitro* and Wss1 inactivation hyper-sensitizes cells to DPC-inducing agents (*e.g*. formaldehyde). Concomitantly, a study using *Xenopus* egg extract reported the existence of a replication-coupled, proteasome-independent, proteolytic mechanism for DPC repair [11]. However, the identity of the protease in metazoans remained elusive until very recently, when several laboratories demonstrated that the DNA-dependent metalloprotease for DPCs is SPRTN (also known as DVC1) [[12], [13], [14], [15], [16]], previously described as a regulator of translesion synthesis following UV damage [[17], [18], [19], [20], [21], [22]]. Like its functional homolog Wss1, human SPRTN is active *in vitro* against several DNA-associated proteins [12,14,15]. SPRTN depletion in *C. elegans*, mouse embryonic fibroblasts (MEFs) and cultured human cells causes hypersensitivity to general (*e.g*. formaldehyde) and specific (*e.g*. camptothecin) DPC-inducing agents [[12], [13], [14], [15]]. However, Wss1 and SPRTN are not orthologs and most of the sequence similarity between the two proteins lies within their N-terminal protease domains (with the conserved metalloprotease active center HEXXH) [8,23].

### 2.1 ACRC (Acidic repeat containing) protein

**Fig. 2:**
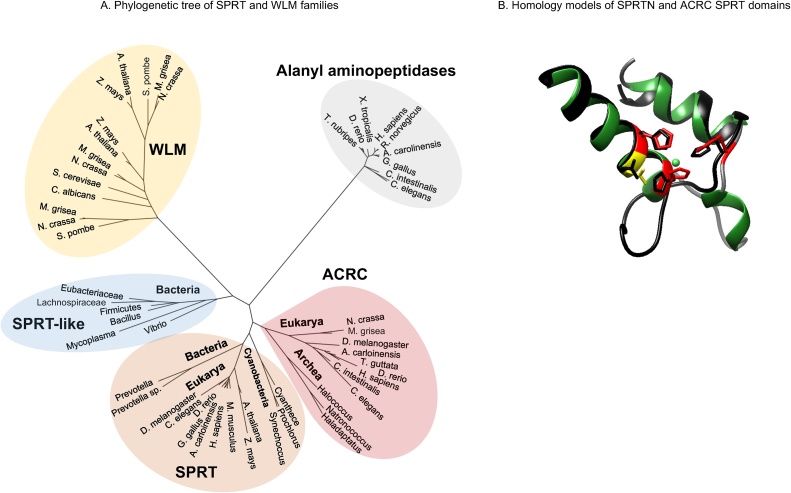
A. Phylogenetic tree of SPRT and WLM families. The newly identified SPRT-like protein group, ACRC, is evolutionary close to the SPRT family. ACRC orthologs are found in archea and eukarya, while absent in prokaryotes. Alanyl aminopeptidase family of gluzincins were used as an outgroup. Protein sequences of SprT and WLM domains were aligned using MAFFT and the phylogenetic tree was constructed in PhyML. B. Comparison of SPRTN and ACRC SprT domains. The protease core of ACRC is similar to that of SPRTN. The SprT domain of ACRC (in black) was modelled according to the yeast Wss1b structure (5JIG) and overlapped with the model of the SprT domain of SPRTN protein (abylysin template, 4JIU) (in multiple colours). Protease core consists of two α-helices (in green), catalytic glutamate (in yellow) and three zinc binding histidines (in red). Homology models were created in SWISS-MODEL workspace and visualized in UCSF Chimera.

A second potential DPC protease in higher eukaryotes might exist. ACRC, also known as GCNA (Germ Cell Nuclear Antigen), was recently identified as a SprT domain containing protein [24,25]. ACRC has eluded the connection to SPRTN proteases until recently, probably due to the prevalence of highly disordered regions within the protein (80–100%), which make it hard to perform accurate sequence alignments, and due to the fact that the mouse ACRC ortholog lacks a SprT domain [24]. Besides mice, the SprT domain is present in all metazoan ACRC orthologs (Fig. 2).

We have performed phylogenetic analysis on multiple sequence alignment of SprT and WLM domains in metazoans using the MAFFT alignment algorithm [26] and Maximum Likelihood analysis (PhyML) [27]. Another family of gluzincins, Alanyl aminopeptidases, was used for comparison of evolutionary distance between ACRC on one side and SPRTN and WLM families on the other. Phylogenetically, ACRC is very close to SPRTN (Fig. 2A), while it is more distant to Wss1 orthologs of the WLM family.

In line with the phylogenetic proximity, the 3D structure of the protease core within the SprT domain of ACRC is very similar to that of SPRTN (Fig. 2B). The putative protease core of ACRC includes two α-helices bearing three zinc-binding histidines and a catalytic glutamate residue which together form a HEXXH motif, a characteristic of all zinc-dependent metalloproteases. The SprT domain of ACRC was modelled according to the yeast Wss1b structure (5JIG) and the SprT domain of SPRTN was modelled according to the abylysin template (4JIU) using the SWISS-MODEL workspace (Fig. 2B).

Given the phylogenetic proximity of SPRTN and ACRC families and high degree of conservation of their protease cores, it will be interesting to determine if ACRC is proteolytically active and whether it plays a role in DPC repair.

## 3 Mechanism of DPC proteolysis repair

DPCs are heterogeneous in the nature and size of crosslinked protein/s. Nevertheless, DPC proteases are capable of digesting proteins of variable size *in vitro*, ranging from histones to topoisomerases, in a DNA-dependent manner. Perhaps not surprisingly, histones and topoisomerases are among the most abundant DPCs in SPRTN-depleted cells [12]. Human SPRTN associates with the replisome and removes DPCs in front of the replication fork [12,16]. Consistently, mammalian non-replicative cells are not sensitive to cross-linking agents [12]. These evidences underscore the essential role of SPRTN in preventing replication stalling upon DPC formation and account for the observed increase in SPRTN levels in S phase (described in more detail below) [17].

Interestingly, a replication-independent function was described for the *Drosophila* SPRTN ortholog MH in male pronuclei before the first zygotic division; this feature has been linked to the high frequency of topoisomerase-dependent DNA topological rearrangements at this developmental stage [25]. This study emphasizes how DPC removal is critical during DNA transactions outside of S phase. A replication-independent function for SPRTN is also suggested by studies in post-mitotic *C. elegans* [15]. While SPRTN levels in G1 phase, albeit low, might be sufficient to sustain DPC repair, it is conceivable that other proteases (*e.g*. other SprT proteases, ACRC), the 26S proteasome or repair pathways operate when cells cannot count on SPRTN-dependent proteolysis. Genetic studies in yeast established that NER can process DPCs independently of DPC proteolysis. This led to a model in which NER removes the bulk of DPCs prior to S phase, while Wss1 or HR are needed to circumvent the remaining, particularly toxic DPCs in S phase [10]. In mammalian cells, however, the contribution of NER to overall DPC removal appears to be negligible [12]. While other pathways have been implicated in DPC repair, a deeper understanding of how they are coordinated with DPCP will require further investigation.

## 4 Regulation of DPC proteases

**Fig. 3:**
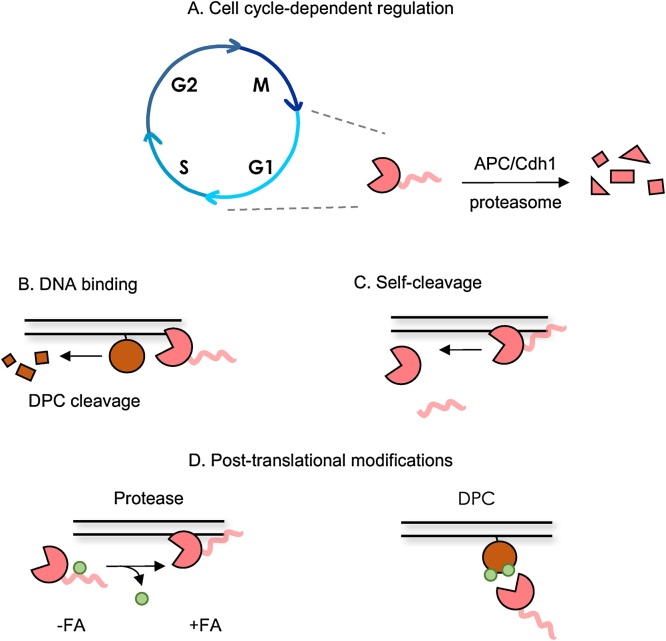
Summary of regulation layers of DPC proteases. A. SPRTN is degraded in G1 phase after ubiquitylation by APC/Cdh1. This ensures the levels of the protease are low when its activity is less needed. B. Wss1 or SPRTN (in red) are recruited to DNA for cleavage of the substrate (DPC; in brown): this ensures that their protease activity is restricted to the chromatin environment. C. DNA also stimulates Wss1 or SPRTN self-cleavage: this helps to prevent unscheduled proteolysis of DNA-associated proteins. D. Left, SPRTN is modified by ubiquitin and de-ubiquitylated upon DPC formation induced by formaldehyde (FA). Right, DPCs might be modified by ubiquitin (or ubiquitin-like proteins) to recruit DPC proteases to the site of damage. Colour Code: Red: Wss1 or SPRTN protease; Brown: DPC; Green: Ubiquitin or Ubiquitin-like protein.

DPC proteases are promiscuous, and their activity is potentially deleterious. Therefore, their activity must be strictly regulated in order to direct the protease to particular cross-linked proteins and to prevent unspecific cleavage of other DNA-bound proteins, such as components of the replisome. Research on Wss1 and SPRTN has so far highlighted four layers of regulation: 1) cell-cycle control of protein levels; 2) DNA binding; 3) self-cleavage and 4) post-translational modifications (PTMs) (Fig. 3).

### 4.1 Cell cycle regulation

Association of SPRTN with the replisome ensures that DPC proteolysis happens as the replication fork runs into DPCs. In further support of a replication-dependent mechanism, SPRTN levels are subjected to cell cycle regulation. SPRTN is predominantly expressed during S phase and G2 and degraded in G1 *via* APC/Cdh1 [17]. The G1 degradation might be necessary to reduce the levels of this promiscuous protease when it is less needed. An analogous cell cycle dependency for Wss1 has not been documented, although its levels are reportedly very low in general [23]. DNA transactions other than replication can be similarly affected by DPCs [1]. Whether other repair pathways or other proteases, especially the 26S proteasome, take over DPC repair outside of S phase is not clear. In particular, a link between DPCP repair and transcription, which is not limited to S phase, has not yet been explored.

### 4.2 DNA binding

A common feature of Wss1 and SPRTN is their DNA-dependent activity: DNA acts as a scaffold to bring enzyme and substrate into close proximity. Wss1 and SPRTN bind DNA *via* one (Wss1) or more (SPRTN) DNA-binding motifs [10,12,14,15,28]. While being an effective strategy to contain proteolytic activity to the chromatin environment, it does not account for how DPC proteases are restrained from processing other essential DNA-associated proteins. The biochemical basis for the promiscuity of DPC proteases could be explained by the recently published structure of *S. cerevisiae* Wss1 protease domain, which shows a solvent-exposed active site lacking a defined substrate-binding cleft [29].

### 4.3 Self-cleavage of the DPC protease

One possible mechanism by which DPC proteases are regulated on chromatin is *via* self-cleavage. For both Wss1 and SPRTN, DNA has been shown to stimulate self-cleavage *in trans* [10,[12], [13], [14], [15],^30^]. Self-cleavage releases C-terminal fragments from the DNA, leaving the protease domain intact. It is unclear whether this has any functional relevance, *e.g.* increased proteolytic activity, as was suggested for Wss1 (Cysteine switch) [30]. More likely, self-cleavage could be a protective mechanism that releases the active proteases from the chromatin to either preserve non-covalently associated proteins or terminate proteolysis after DPC removal. Consistent with this model is the observation that the auto-cleavage products of SPRTN cannot bind DNA [15]. Importantly, self-cleavage might partially explain how cells preserve functional DNA-associated proteins from proteolysis, but does not clarify how SPRTN substrate specificity is achieved. Thus, other regulatory mechanisms must exist.

While self-cleavage is stimulated *in vitro* by both single- and double-stranded DNA [10,12,14,15,30], proteolytic processing of substrates might be preferentially fostered by single-stranded DNA [15]. In line with this observation, single-stranded DNA forms during replication whenever the replicative polymerase stalls behind a DPC while the helicase progresses past the lesion [1]. However, this model does not account for those large DPCs that will block helicase progression as well. Therefore, while intriguing, this model is not definitive and disagrees with studies showing that dsDNA and ssDNA are both equally effective in stimulating substrate cleavage and auto-cleavage [[12], [13], [14]]. Thus, more work is needed to explain how DPC proteases are activated when the replication fork encounters a DPC.

### 4.4 Post-translational modifications

SPRTN is mono-ubiquitylated and its recruitment to the chromatin coincides with its de-ubiquitylation [15]. This so-called ubiquitin switch model predicts that SPRTN is kept in an ‘inactive’ conformation by virtue of the interaction between the SPRTN ubiquitin-binding domain (UBZ) and the modifying ubiquitin; deubiquitylation by a so-far elusive deubiquitinating enzyme would thus ‘activate’ SPRTN upon DPC formation.

In addition to ubiquitylation, several screening studies have identified numerous SUMOylation sites on SPRTN. Notably, modification by SUMO is increased after stress is applied (*e.g*. proteasomal inhibition or replication stress) [[31], [32], [33], [34]]. Thus, it is tempting to speculate that cellular stress and/or DNA damage trigger PTMs that activate SPRTN.

On the other hand, PTMs could be a way to direct DPC proteases to their substrates or sites of damage. In *Xenopus* egg extract DPC processing requires free ubiquitin [11]. This raises the intriguing possibility that ubiquitin labels DPCs for SPRTN recruitment. Some groups have shown that SPRTN binds ubiquitylated PCNA (Rad18 pathway) at stalled replication forks after UV damage [[19], [20], [21], [22]]. Although this observation remains controversial [17,18], genetic data would suggest that a similar (Rad18-dependent) mechanism applies to DPC-dependent damage [14]. Consistently, Rad18-mediated PCNA ubiquitylation also occurs upon accumulation of ssDNA at stalled replication forks [35,36].

The regulation by PTMs might differ in lower eukaryotes, where SUMO rather than ubiquitin might recruit the DPC protease to the site of protein crosslinks. Yeast Wss1 binds SUMO *via* two C-terminal SUMO-binding motifs (SIMs) which are required for resistance to formaldehyde [10]. The fact that Wss1 and SPRTN might be subjected to different modes of regulation is also suggested by the experimental evidence that plasmid-borne SPRTN cannot rescue the camptothecin sensitivity of a yeast *wss1 tdp1* double deletion mutant [13].

Overall, it is becoming clear that DPC protease activity is subjected to several layers of regulation; however, the regulatory modes are far from being completely deciphered and are a matter of future investigations.

## 5 DPC proteolysis repair of Top1- & Top2-ccs

Perhaps the most biologically and therapeutically relevant DPCs are Topoisomerase 1 and 2 cleavage complexes (Top-ccs). Upstream proteolysis of Top-ccs by DPC proteases has emerged as an important component of Top-cc repair [12,16]. When topoisomerases cleave DNA to resolve topological stress they form a catalytic intermediate, known as a Top-cc, in which a tyrosine in their active site is covalently bound to the phosphate group of a nucleotide. Top1 cleaves one strand of DNA, and swivels the broken DNA strand around the unbroken strand before re-ligation. Top1 can also form double-strand breaks (DSBs) if it cleaves opposite a DNA lesion or if a Top1-cc is encountered by a replication fork (leading to a single-ended DSB) [37]. Top2, meanwhile, always generates DSBs and re-ligates both DNA strands. Top-ccs are normally transient but can become trapped when a topoisomerase cleaves near DNA alterations (*e.g.* nicks, breaks, abasic sites) or upon exposure to endogenous or exogenous crosslinking agents [6].

Top-ccs disrupt essential DNA processes, including DNA replication and transcription, and can therefore have pathogenic or cytotoxic consequences. For example, both Top1- and 2-ccs are associated with neurodegenerative disorders [38,39]. Furthermore, the widely-used anti-cancer drugs, camptothecin (CPT) and etoposide (ETO), kill cancer cells by trapping Top1- and 2-ccs, respectively. ETO can also induce chromosomal translocations associated with secondary malignancies, demonstrating the tumorigenic potential of Top-ccs [40,41].

Top-ccs were previously known to be repaired by two different pathways operating on the DNA adjacent to or linked to the trapped cleavage complex, *i.e.*:

1) **Excision by the phosphodiesterases** TDP1 and TDP2: TDP1 and TDP2 hydrolyse the phosphodiester bonds linking Top-ccs to DNA. TDP1 and TDP2 preferentially resolve Top1- and 2-ccs, respectively.
2) **DNA cleavage by endonucleases**: Top-ccs can be removed by endonucleases that cleave the DNA flanking a Top-cc. Many nucleases have been implicated in this process, including XPF-ERCC1 (for Top1-ccs), the MRN complex, and CtIP [[42], [43], [44]].

However, a novel mechanism of Top-ccs repair has been identified:

3) **DPC proteolysis repair** has recently emerged as a key component of the response to Top-ccs. Various lines of investigation posited that such a mechanism must exist, primarily to allow phosphodiesterases access to the phosphotyrosyl bond concealed inside a Top-cc. Indeed, TDP1 and TDP2 resolve Top-ccs *in vitro* after the Top-cc is subjected to heat denaturation or proteolytic digestion in most cases [[45], [46], [47]].

A role for proteases in processing the bulk of the protein components of Top-ccs was first demonstrated in yeast [10]. It was found that cells lacking TDP1 relied on the protease activity of Wss1 for their survival, especially in the presence of CPT. SPRTN counteracts Top1- and 2-ccs even in the absence of exogenous DPC-inducing agents and SPRTN depletion alone hypersensitizes cells to both CPT and ETO [12]. Notably, hypomorphic SPRTN mice accumulate Top1ccs, particularly in the liver, and develop liver tumours at an early age [16]. Whereas Wss1 and TDP1 act in distinct pathways to repair Top1ccs, SPRTN appears to act upstream of TDP1, at least in human cells [12]. The 26S proteasome is also likely to be involved in Top1/2 processing, however, *in vivo* its contribution is usually only observed after treatment with high doses of CPT and ETO [46,48,49].

The question of how DPCs are distinguished from essential proteins that are tightly bound to chromatin has not been fully addressed, but, at least in Top-cc repair, post-translational modifications are proposed to drive this distinction. Both Top1- and 2-ccs are extensively ubiquitylated and SUMOylated in both yeast and humans and both of these modifications are induced by treatment with Top1- or Top2-specific poisons [[50], [51], [52], [53], [54]].

Initial indications that SUMO might initiate Top-cc repair came from yeast which were hypersensitive to CPT when the E2 SUMO conjugating enzyme/ligase, Ubc9, was deleted [50]. In mice and humans, ATM regulates the SUMO/ubiquitin-dependent turnover of Top1ccs, and Top1cc accumulation may contribute to the neuropathology of ataxia telangiectasia patients [55,56]. In yeast, both Top1- and 2-ccs are subject to SUMOylation by E3 SUMO ligases of the PIAS family and are then ubiquitylated by SUMO-targeted ubiquitin ligases (STUbLs) [57,58]. For both Top1- and 2-ccs, this ubiquitylation is thought to recruit Cdc48 (p97 in humans), a hexameric ATPase capable of unfolding substrates, thereby facilitating their removal from chromatin and promoting their degradation by the 26S proteasome [57,59].

A recent report has placed further emphasis on the role of SUMO in the upstream processing of Top-ccs [60]. The E3 SUMO ligase ZNF451 modulates proteasome-independent Top2cc repair *via* two mechanisms. Firstly, by directly binding Top2-ccs, ZNF451 induces a conformational change that facilitates TDP2′s access to the phosphotyrosyl bond that links Top2 to DNA. Secondly, ZNF451 conjugates SUMO-2/3 chains to Top2-ccs, which serve as a signal for the recruitment of TDP2. Further studies will hopefully address the interplay and relative contribution of each repair pathway and PTM to counteracting Top-cc-induced genome instability.

It seems plausible that PTMs could be important either for recruiting DPC proteases or for stimulating their protease activity *in vivo*. Indeed, Wss1, SPRTN and ACRC all possess SIMs [24]. Wss1 variants which cannot bind SUMO fail to fully rescue the viability of Wss1-deficient cells [10]. Recent reports suggest that SPRTN’s UBZ, while required for its role in the response to UV-induced damage, is not necessary for DPC proteolysis [[15], [16], [17], [18]].

Furthermore, both Wss1 and SPRTN have motifs that enable them to interact with p97. Wss1 requires its interaction with Cdc48 to counteract CPT-induced toxicity [10]. Cdc48 also promotes the degradation of SUMO/ubiquitylated Top2-ccs *via* the 26S proteasome [57]. On the other hand, SPRTN’s protease domain alone can rescue the DPC repair defects of SPRTN deficient cells [15,16]. However, these experiments tend to involve overexpressing SPRTN’s protease domain which could obscure the role of other domains (*e.g.* SHP, and thus p97) in the subtle fine-tuning of SPRTN activity.

As mentioned above, SPRTN expression is mainly restricted to S phase and coupled to replication [12]. Wss1 also apparently acts on DPCs that have escaped repair by NER and entered S phase. However, DPCs are also potentially very toxic in other cell cycle stages, due for example to interference with transcription, as demonstrated by the neuronal disorders resulting from TDP1 and TDP2 mutations. It is therefore likely that there exist other factors acting upstream of TDP1 and TDP2 in different phases of the cell cycle.

## 6 DPC proteolysis repair in disease & cancer therapy

### 6.1 DPCP repair defect causes accelerated ageing and liver cancer

In humans, *SPRTN* mutations cause a medical condition known as SPARTAN syndrome or Ruijs-Aalfs syndrome (RJALS) characterized by premature aging, early onset hepatocellular carcinoma and chromosomal instability [61,62]. RJALS patient-derived cells display an accumulation of DPCs and hypersensitivity to DNA-protein crosslinking agents, along with DNA replication stress and an increased frequency of DSBs [12,13,61]. These defects can be reproduced in cultured cells upon ectopic expression of disease-associated SPRTN mutants variants [12,61]. SPRTN knock-out in mice is embryonic lethal, but conditional knock-out in MEFs causes replication defects and genomic instability [63]. Additionally, SPRTN hypomorphic mice recapitulate RJALS patient phenotypes, namely progeroid phenotypes and liver tumours [16]. Overall, these phenotypes establish an unequivocal link between DNA replication-coupled DPC removal and protection from accelerated ageing and cancer.

### 6.2 Therapeutic potential for intervention on DPCP repair

The toxic potential of DPCs is exploited in cancer therapy, where drugs that induce DPCs are already widely used to kill cancer cells. Indeed, nearly half of all currently-used anti-cancer regimens consist of drugs that trap Top-ccs [64]. Camptothecins bind the interface between the DNA and Top1: they trap Top1ccs as soon as they form, preventing re-ligation of the broken DNA strand. Camptothecins are effective against a variety of different types of cancers and are routinely used to treat metastatic colorectal cancer [65]. A new class of non-camptothecin derived compounds, the indenoisoquinolines, are also interfacial Top1cc inhibitors but exhibit many improved features. For instance, they are less rapidly metabolised by cells than camptothecins and, unlike camptothecins, they induce Top1ccs which persist even after drug withdrawal. Indenoisoquinolines are showing promising results in clinical trials for the treatment of solid tumours and lymphomas [66] (http://clinicaltrials.gov/show/NCT01245192).

Most clinically-used Top2-targetting drugs trap Top2-ccs, including etoposide, doxorubicin (and other anthracyclines), and mitoxanthrone, but do so *via* different mechanisms. For example, doxorubicin intercalates in DNA and stabilises Top2ccs, whereas etoposide is specific for the Top2-DNA interface. Cancers with Top2a gene amplifications (*e.g*. Her2-positive breast cancers) often exhibit enhanced sensitivity to various Top2 poisons [40].

As acquired resistance to topoisomerase-trapping drugs is common, much attention has been placed on targeting factors which modulate sensitivity to these drugs. High-throughput screens have identified many promising hits such as those which inhibit TDP1 by mimicking its phosphotyrosine substrate [67]. These inhibitors are likely to be of significant value in cancers, such as non-small cell lung cancers (NSCLCs), where TDP1 is reported to be overexpressed [68]. Deazaflavin inhibitors of TDP2 also show promising selectively and potency in pre-clinical trials [69].

The covalent trapping of other DNA enzymes underlies the effectiveness of other chemotherapeutic agents, such as 5-aza-2′-deoxycytidine (5-aza-dC), which is used to treat myelodysplastic syndromes and acute myeloid leukaemia [70]. 5-aza-dC is a cytosine analogue that is incorporated into DNA. While attempting to methylate the 5-aza-dC molecule, DNMT1 is trapped leading to loss of global DNA methylation and cell death [71].

DPC induction is increasingly being recognized as an important mode of action for other anti-cancer drugs that are already in clinical use. For example, one way in which platinum-based agents, such as cisplatin and oxaliplatin, exert their cytoxicity is by inducing protein crosslinking to platinum-DNA complexes. This includes the crosslinking of histones, but also of potentially any protein in the vicinity of DNA. Notably, there is some evidence indicating a positive correlation between the clinical efficacy of platinum compounds and the extent to which they induce DPCs [72,73]. Another example is PARP trapping by PARP inhibitors. The effectiveness of PARP inhibitors (PARPi) has been demonstrated to correlate better with their ability to trap PARP on DNA than it does with their ability to inhibit PARP catalytic activity [74]. An understanding of how PARP-DNA complexes and protein-platinum-DNA complexes are resolved could help improve treatment for PARPi/Platinum-resistant breast and ovarian cancers as well as other PARPi resistant cancers.

Targeting DPC repair pathways could be beneficial for cancer therapy with currently ineffectual treatment regimens. For example, inhibitors of DPC repair could sensitize hypoxic tumours following the IR treatment, given that IR mainly induces DPC formation in hypoxic tissues [75].

## 7 Conclusion and perspectives

The emergence of DPC proteases has exciting implications for cancer therapy and ageing. The importance of these enzymes will stimulate further work into their physiological roles and modes of regulation. In particular, it will be important to address how their promiscuous activity is targeted to specific substrates and how DPC repair pathway choice is made. Structural insights into SPRTN substrate binding and specificity could facilitate the development of drugs that target its protease activity. While the contribution of DPCs to carcinogenesis has attracted much attention, their role in ageing requires further elucidation. Unravelling these questions will have significant ramifications for our understanding human diseases and the development of effective therapies.

## Conflict of interest

There are no conflicts of interest to declare.
